# Recent Progress of Chitosan and Chitosan Derivatives-Based Nanoparticles: Pharmaceutical Perspectives of Oral Insulin Delivery

**DOI:** 10.3390/ph13100307

**Published:** 2020-10-14

**Authors:** Salma Seyam, Norsyafikah Asyilla Nordin, Mulham Alfatama

**Affiliations:** Faculty of Pharmacy, Universiti Sultan Zainal Abidin, Besut Campus, Besut 22200, Terengganu, Malaysia; salma4131@gmail.com (S.S.); asyillanordin@unisza.edu.my (N.A.N.)

**Keywords:** chitosan, oral insulin delivery, nanoparticles, diabetes mellitus, ionic gelation

## Abstract

Diabetes mellitus is a chronic endocrine disease, affecting more than 400 million people around the world. Patients with poorly controlled blood glucose levels are liable to suffer from life-threatening complications, such as cardiovascular, neuropathy, retinopathy and even premature death. Today, subcutaneous parenteral is still the most common route for insulin therapy. Oral insulin administration is favourable and convenient to the patients. In contrast to injection route, oral insulin delivery mimics the physiological pathway of endogenous insulin secretion. However, oral insulin has poor bioavailability (less than 2%) due to the harsh physiological environment through the gastrointestinal tract (GIT). Over the last few decades, many attempts have been made to achieve an effective oral insulin formulation with high bioavailability using insulin encapsulation into nanoparticles as advanced technology. Various natural polymers have been employed to fabricate nanoparticles as a delivery vehicle for insulin oral administration. Chitosan, a natural polymer, is extensively studied due to the attractive properties, such as biodegradability, biocompatibility, bioactivity, nontoxicity and polycationic nature. Numerous studies were conducted to evaluate chitosan and chitosan derivatives-based nanoparticles capabilities for oral insulin delivery. This review highlights strategies that have been applied in the recent five years to fabricate chitosan/chitosan derivatives-based nanoparticles for oral insulin delivery. A summary of the barriers hurdle insulin absorption rendering its low bioavailability such as physical, chemical and enzymatic barriers are highlighted with an emphasis on the most common methods of chitosan nanoparticles preparation. Nanocarriers are able to improve the absorption of insulin through GIT, deliver insulin to the blood circulation and lower blood glucose levels. In spite of some drawbacks encountered in this technology, chitosan and chitosan derivatives-based nanoparticles are greatly promising entities for oral insulin delivery.

## 1. Introduction

Diabetes mellitus (DM), one of the major epidemics worldwide of the 21st century, is a chronic disease that developed in about 451 million people in 2017 and this number is anticipated to increase to 693 million by 2045 worldwide [1,2]. To date, subcutaneous injections remain the conventional way to deliver insulin daily. However, this route is associated with several drawbacks including poor patient compliance as a result of needle fears, allergic reactions, pain and hypoglycemic episodes [3]. Oral insulin delivery, on the other hand, has been the research of interest globally for decades. Effective oral insulin dose must survive along the gastrointestinal tract (GIT), cross the mucus layer, transport through the intestinal epithelial cells, enter the liver via portal vein and finally reach the systemic circulation [4]. However, mere oral administration of insulin is encountered with enzymatic and physiological barriers that negate insulin absorption through intestinal epithelial cells. Such hurdles render insulin poor oral bioavailability, despite the oral route is the most favourable mode of diabetes management [1].

**Figure 1:**
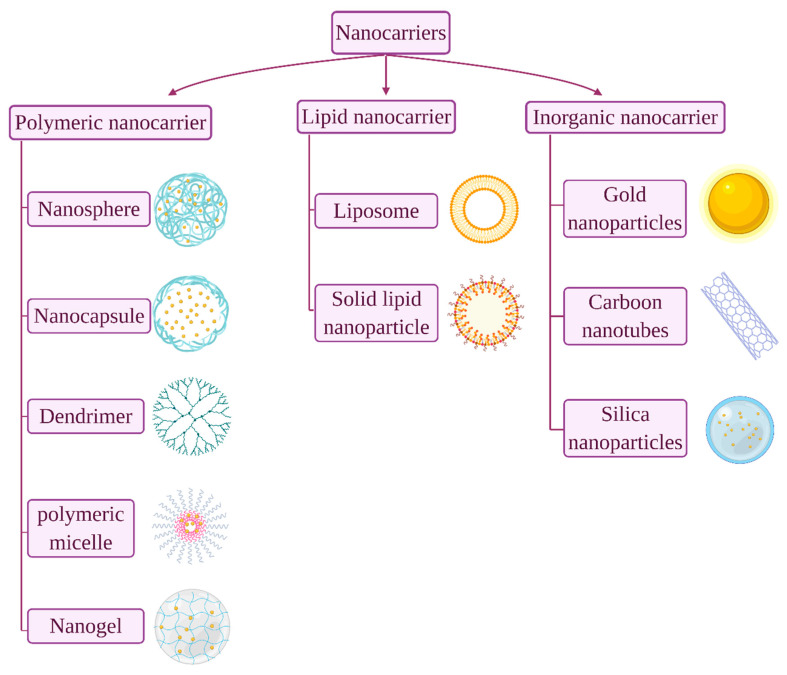
Common nanocarriers used for oral protein/peptide delivery.

In order to circumvent the above mentioned challenges, numerous studies have been carried out to develop efficient oral insulin delivery systems where nanotechnology appeared to be a favourable platform. Currently, the application of nanomaterials attracts wider attention in pharmaceutical and biomedical research. Nanoparticulates are defined as entities that are synthesized using nanomaterials that endow unique functionality to the delivery system. The drug content and release profiles of nanosystems are tailorable simply by modulating their starting material composition and physical traits [5]. Generally, nanocarriers can be classified according to their compositional structure into polymeric nanoparticle, lipid-based nanoparticles and inorganic nanoparticle (Figure 1) [6]. In the last two decades, great interest was granted to polymeric and lipid-based nanoparticles over inorganic metal ones for proteins/peptides oral delivery, owing to their biocompatibility and biodegradability, as well as promising clinical outcomes [7,8]. Polymeric nanoparticles being inert and non-immunogenicity, it enables them to escape from endosomal recognition and avoiding of degradation by lysosomes [6]. Moreover, while nanoparticles generally facilitate insulin transportation in the intestine by both transcellular and paracellular pathways, polymeric nanoparticles significantly enhance insulin absorption through paracellular pathway by reversely opening the tight junctions between adjacent cells [9]. Thus, of all the nanoparticle used in drug delivery designs, polymeric nanoparticles have gained great interest. Furthermore, methods of formulation are widely available therefore, the range of applications has been expanding to include variety of hydrophilic and hydrophobic dugs of chemical drug classes and dosage forms [10,11]. The smart nanocarriers, synthesized from stimulus-responsive building blocks as part of a polymeric structure, can be controlled to release drugs in response to environmental stimuli such as temperature and pH. In addition, nanocarriers can be decorated with targeting ligand for site-specific drug delivery [12]. Polymeric nanoparticles can be either synthesized from biodegradable synthetic polymers, such as poly(lactide-glycolide) (PLGA) copolymers, polyacrylates, or from natural polymers, such as chitosan, alginate, collagen and albumin. Notable advantages of the natural polymeric-based nanoparticles render them particularly unique due to their abundance in nature, non-toxic with established safety profile and easily modifiable [13].

Among all polysaccharides, chitosan has been the primary interest for many investigators in the designing of oral drug delivery system as a function of its biodegradable, biocompatible, smooth of processing and its digestibility by colonic microbial enzymes to emerge colon-targeted delivery of drugs [14]. Nanoparticles-based chitosan are particularly favourable for the mucosal route due to low toxicity, tunable physiochemical properties and mucoadhesion. There are several methods to formulate chitosan nanoparticles, such as ionic gelation, polyelectrolyte complexation, reverse micellar, emulsion solvent diffusion and electrospraying techniques [15]. Careful selection of nanoparticles composition and method of preparation is essential to meet the objectives of protecting the encapsulant (insulin) and deliver it in a sufficient manner to the blood circulation hence, improve its bioavailability. Thus, the nanoparticle formulator must precisely match the desired chemical and physical attributes of chitosan with reference to the biological environment, with chitosan processing technique [10].

What makes chitosan unique over other polysaccharides for oral drug delivery is its chemical structure that allows specific modifications through modulation in the chitosan amine or hydroxyl functional groups [16]. With regards to pharmaceutical applications, these chemical moieties can be utilised to conjugate drugs directly or via linkers. Abundance of amino groups on the backbone of chitosan would enable any amine related conjugations with other molecules, such as methacrylation [17] and carboxymethylation [18].

This review will discuss how recent developments in chitosan/chitosan derivatives-based nanotechnology have been emerged in a multitude of platforms for safe and efficient delivery of insulin orally for the treatment of DM.

### 1.1. Diabetes Mellitus

Diabetes mellitus is a chronic endocrine disease in which an elevation of blood glucose level occurs as a result of reduced or inability of pancreas to produce insulin or due to peripheral tissue uptake defects of insulin [19]. Diabetes can primarily be classified into two types: type 1 diabetes mellitus (T1DM) and type 2 diabetes mellitus (T2DM). In T1DM, the pancreas terminates or reduces insulin production due to pancreatic β-cell destruction, whereas in T2DM, the cells manifest low sensitivity towards insulin and consequently both types lead to hyperglycemia [20]. Poorly controlled blood glucose levels can bring about serious adverse effects in cardiovascular system, nervous system, retina, and even early death [21,22], hence exogenous insulin intake is imperative in patients with T1DM and advanced T2DM [23].

### 1.2. Insulin

**Figure 2:**
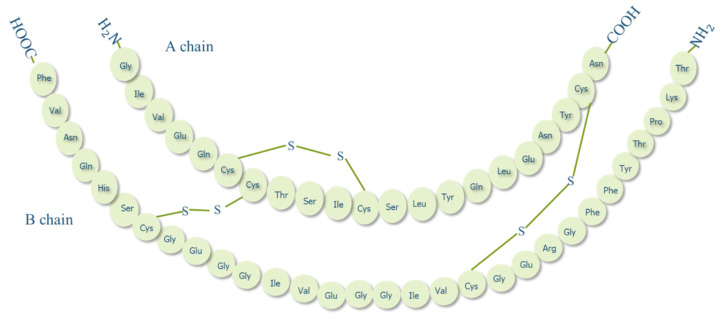
Structure of human insulin.

Insulin is an anabolic polypeptide hormone, synthesized in high amounts by islets of Langerhans of pancreatic β-cells and is responsible to maintain blood glucose level at normal ranges. Proinsulin (an insulin prohormone precursor) is composed of three domains: an amino-terminal B chain, a carboxy-terminal A chain, and a connecting peptide in the middle denoted by C-peptide. By the cleavage of C-peptide, insulin is formed as a quaternary macromolecule composed of two polypeptide chains, A chain (21 amino acid residues) and B chain (30 amino acid residues) that are linked by disulphide bonds (Figure 2) [24]. In 1922, insulin was first successfully isolated by a team of Canadian scientists in Toronto; a discovery that brought about a true medical success and a milestone in the history of treating diabetes [25]. While insulin has been available for treating diabetes for almost a century now, to date, the most common insulin therapy is to be administered to diabetic patients through the parenteral route. Even though this route is still the best route in terms of effectiveness, insulin administered subcutaneously is delivered directly to the peripheral circulation, unmet the endogenous insulin pathway [26]. As a result, insulin enters the liver, which is the main target organ of insulin, at a much lower concentration than normal endogenous insulin which may give rise to hyperinsulinemia, weight gain, and hypoglycemic risks [27]. Moreover, injection is invasive and may induce local tissue necrosis, infection, and allergy that in long-term treatment may lead to low patient compliance and serious complications such as, nerve damage, insulin resistance and hypokalemia [28]. Therefore, alternative routes for insulin delivery were widely investigated recently, such as pulmonary, nasal, buccal, transdermal and oral [29].

### 1.3. Oral Insulin Delivery

**Figure 3:**
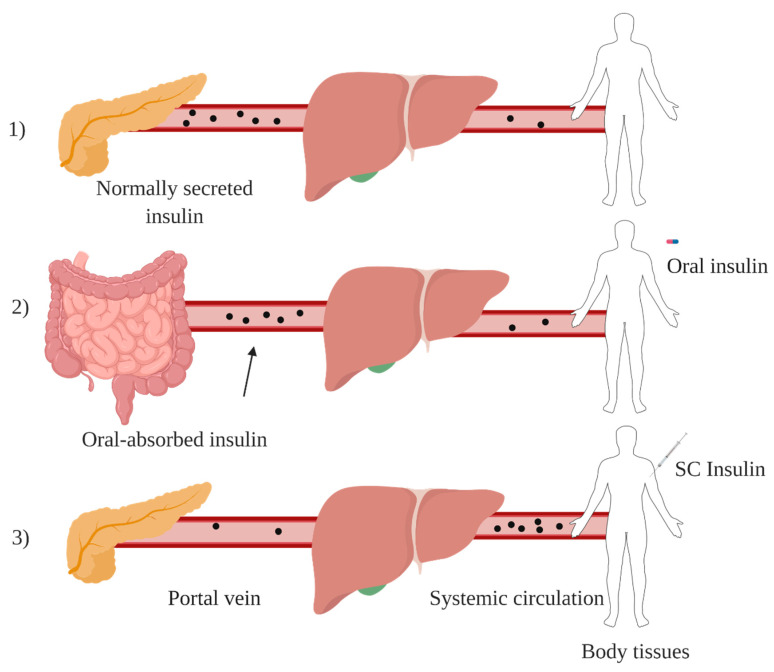
Endogenous insulin pathway under normal physiology (**1**), orally administered insulin pathway (**2**) and injected insulin pathway in diabetic patients (**3**). Created with BioRender.com.

Among all alternative routes for insulin administration, oral route is the most favourable approach and mimics the endogenous insulin pathway. After oral administration, insulin is absorbed from intestinal lumen and transported via portal circulation to the liver in which the first pass effect takes place, generating a high porto-systemic gradient (Figure 3). Insulin then reaches peripheral circulation at relatively low levels, imitating physiological insulin pathway and avoiding side effects associated with subcutaneous route such as, hypoglycemic episodes and weight gain [30]. However, insulin oral administration is usually characterised by poor bioavailability (<2%) [31], due to enzymatic degradation, low stability at different pHs and low permeability of GIT [32].

### 1.4. Barriers to Oral Insulin Delivery

The development of oral insulin delivery system is associated with persistent physiological challenges, where GIT is envisaged to be the main barrier. GIT is responsible for digesting food and selectively absorbing nutrients, electrolytes and fluids. Concurrently, GIT confers a protective barrier against toxic materials such as peptides, viruses and bacteria [33]. These functions are accomplished by a layer of neighboring absorptive and secretory cells, tight junctions that narrowing the paracellular spaces between epithelial cells, a viscous layer of mucus, different pH conditions along GIT and the presence of enzymes involved in food digestion [34]. Table 1 represents a summary of these conditions or barriers that hinder oral delivery of insulin and limit its bioavailability.

### 1.5. Chitosan

**Figure 4:**
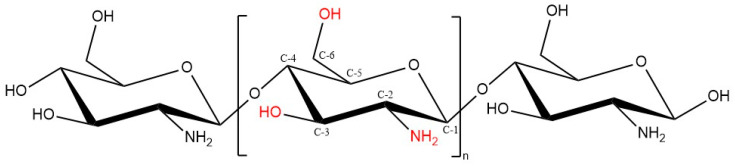
Chitosan chemical structure (DD ≥ 50%).

Chitosan and chitin have received immense attention in different fields in both research and industrial areas, not only because of their biocompatibility, biodegradability and non-toxic properties, but also because they are readily available, inexpensive and environment-friendly biopolymers [41]. Chitin, the second next to cellulose known as the most abundant natural polysaccharides, is a linear polymer comprises of ß-1,4-linked N-acetyl-D-glucosamine, found in the exoskeleton of insects and crustaceans like crab and shrimp, as well as cell walls of fungi [38]. Chitin can be found in diverse degrees of acetylation, in which the degree of acetylation (DA) equals 50% or lower, chitin becomes soluble in acidic aqueous solution and is called chitosan [42]. Chitosan, which is the most valuable derivative of chitin, can be obtained by three different chitin-deacetylation methods: chemical alkaline, microbial and enzyme-based method [43]. Structurally, chitosan is a linear polymer composed of randomly distributed β1-4 linked D-glucosamine and N-acetyl D-glucosamine units, with one amino group (NH_2_) at C-2, and two hydroxyl (OH) groups at C-3 and C-6 in each repeating glycosidic units (Figure 4) [44,45].

Chitosan has received widespread interest to deliver therapeutics for different diseases due to its unique attributes including biocompatibility, biodegradability, mucoadhesion, absorption-enhancing effect, low immune reactions, simplicity of functional groups grafting and non-toxicity [46]. Chitosan also possesses diverse biological activities, such as antioxidant [47], antimicrobial [48], anticancer [49] and wound healing propensity [50]. This advocates the intensive research studies of chitosan and its derivatives for various pharmaceutical and medical applications including tissue engineering [51], food technology [52], wound healing [50], gene delivery [53] and textile industry [54]. Besides, it has also been widely employed in various forms in drug delivery systems such as tablets [55], microspheres [56], nanoparticles [57], nanofibers [58], beads [59], films [60], hydrogels [61], conjugates [62] and chitosan-based nanocomposites [63].

Chitosan has an essential polycationic property ascribing to its pKa value of 6.5; in acidic solutions the amino groups (NH_2_) on the backbone of chitosan get protonated and become positively charged (NH_3_^+^) making chitosan soluble in aqueous acidic solutions; whereas in an alkaline environment, the amino groups lose their positive charges and chitosan renders insoluble [64]. The physiochemical properties of chitosan including viscosity, solubility, adsorption on solids, elasticity, tear strength and biofunctional propensity are greatly attributed to the molecular weight (MW) and degree of deacetylation (DD). The average MW and DD of chitosan may range from 50 to over 2000 kD, and from 30% to 95%, respectively, depending on the source of chitosan and its preparation method from chitin. Other processing conditions may also alter the physical characteristics and performance of the final chitosan product, such as the type and concentration of reagents, time and temperature used throughout the synthesis process [63,65]. Despite of all the functional properties, chitosan poor solubility hinders its applicability as a result of uneven distribution of acetyl groups and aggregates [66]. This intervenes with the biomedical avails of chitosan, especially at near physiological pH 7.4, where chitosan is insoluble and less effective as an absorption enhancing agent [65,67]. Hence, it is imperative to modulate chitosan solubility via introducing various derivatives to maximise its applications especially in drug design and drug delivery, such as chitosan-based nanoparticles.

### 1.6. Chitosan Nanoparticles

Nanoparticles are defined as entities with the size range from 10 to 999 nm. Different types of insulin-loaded nanoparticles for oral delivery administration have been formulated using various materials such as lipids, metals, proteins, natural polymers and synthetic polymers, either alone or combined [68]. Nanoparticles are advantageous due to their small size, large surface area to volume ratio by which their retention time to reach the intestinal absorption sites are prolonged, thus improved permeation and bioavailability. This in turn reduces the frequency and doses of encapsulant and enhances patient compliance [69].

Over the last few decades, different natural polymers have been adopted to ease the limitations associated with oral insulin absorption exploiting advanced nanotechnology. Chitosan and its derivatives are considered as an excellent choice and have been widely investigated in oral insulin designs. The involvement of chitosan as insulin carrier is based on the mucoadhesive property and its ability to reversibly open the tight junctions of epithelial cells. These properties are mainly attributed to the positive moieties possessed by chitosan surface amine groups [70].

Nanoparticles prepared from chitosan and/or chitosan derivatives generally possess a positively charged surface. Chitosan can be tailored to meet a specific goal owing to its unique functional groups, making it a polymer with a formidable range of potential applications [10]. Chitosan nanoparticles can be synthesised using different methods including ionic gelation, emulsion solvent diffusion, emulsion-droplet coalescence, polyelectrolyte complexation, reverse micelle formation, complex coacervation or solvent evaporation methods.

## 2. Preparation Methods of Chitosan Nanoparticles

Chitosan nanoparticles have been documented in the literature as a potential carrier for various drug delivery. A summary of the most common preparation methods of chitosan nanoparticles is outlined below.

### 2.1. Ionic Gelation

**Figure 5:**
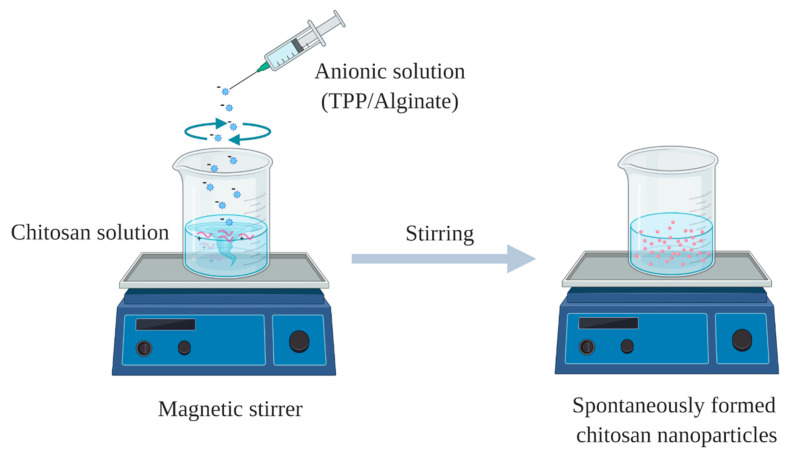
Schematic diagram of chitosan nanoparticles prepared via ionic gelation/polyelectrolyte complexation.

Ionic gelation method was first reported by Calvo et al. [71]. Firstly, chitosan and anionic crosslinker solutions are prepared by dissolving chitosan in diluted acetic acid, while an anionic crosslinker such as sodium tripolyphosphate (TPP) is dissolved in distilled water. Then, ionic gelation takes place by adding TPP solution dropwise into the cationic solution of chitosan under mechanical stirring at room temperature. Nanoparticle will be instantly formed as a result of the complexation between TPP as a negatively charged polyanion, and the positively charged amine group of chitosan by electrostatic forces (Figure 5) [72]. The advantages of this technique include, simple and easy of preparation, organic solvent-free, and can be done under mild conditions, while the possible disadvantage is the relatively higher polydispersity index of the prepared nanoparticles.

### 2.2. Polyelectrolyte Complex (PEC)

Polyelectrolyte complexes are formed by self-assembly of the positively charged chitosan and negatively charged polyelectrolyte macromolecules such as dextran sulphate, alginate, hyaluronic acid and DNA. Nanoparticles will be spontaneously formed upon addition of anionic solution into acetic acid solution of chitosan, under mechanical stirring at room temperature (Figure 5). The advantages of this method are similar to those of ionic gelation technique, however, optimisation of the ratio between oppositely polyelectrolytes is challenging.

### 2.3. Reverse Micellar Method

**Figure 6:**
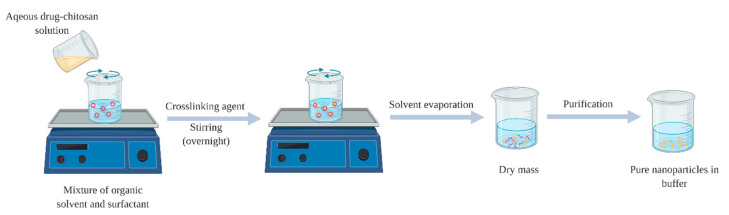
Schematic diagram of chitosan nanoparticles prepared via reverse micellar method.

This method was described by Brunel et al. [73], where the formation of the nanoparticles takes place in an aqueous core of a reverse micellar droplets, followed by cross-linking with glutaraldehyde [74]. Formation of reverse micelles is aided by adding surfactant to the organic solvent, while chitosan-drug aqueous solution with glutaraldehyde as crosslinker should be added with continues stirring to avoid turbidity. In order to attain complete cross-linkage for chitosan, it is advised to maintain the stirring overnight. Then, the organic solvent is evaporated, and the yield nanoparticles can be harvested by precipitation with a suitable salt (Figure 6) [75]. The advantages of this method are associated with the ability of production of small particle size with a narrow size of distribution [76], while the disadvantages including the laborious and time-consuming process and the presence of organic solvent and surfactant.

### 2.4. Emulsion Solvent Diffusion

**Figure 7:**
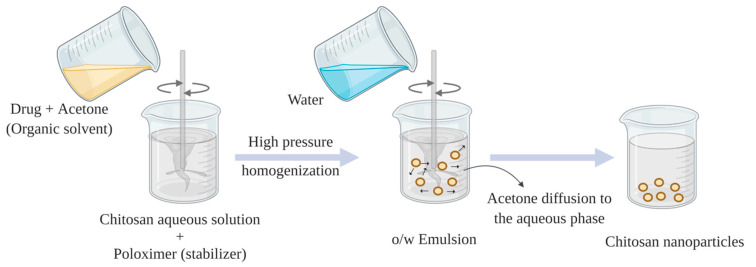
Schematic diagram of chitosan nanoparticles prepared via emulsion solvent diffusion.

This method was first reported by El-Shabouri et al., with principle of partial miscibility of organic solvent as an oil phase with water [77]. Oil-in-water (o/w) emulsion is prepared by pouring organic phase such as methylene chloride or acetone solution of hydrophobic drug into chitosan aqueous solution that contains stabilizer (poloxamer or lecithin) under continuous stirring. Under intense homogenization, the emulsion has to be diluted with a large amount of water to promote a complete diffusion of organic solvent into aqueous phase, overcoming the organic solvent miscibility in water. Finally, nanoparticles are formed by means of polymer (chitosan) precipitation as a result of reduced chitosan solubility as acetone diffuses into the aqueous phase (Figure 7) [75,78]. The advantages of the method are the feasibility to scale up and suitability for hydrophobic drugs encapsulation, while the possible disadvantages can be the use of organic solvent, surfactant and high shear force (vortex).

### 2.5. Electrospraying Technique

**Figure 8:**
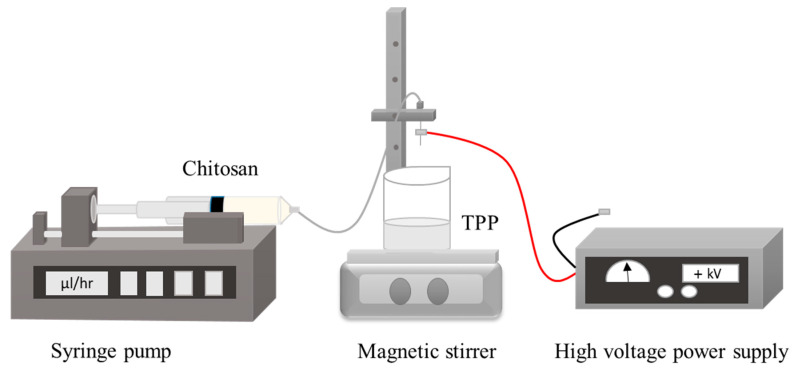
Schematic diagram of chitosan nanoparticles prepared via electrospray technique.

Among various reported nanoparticle preparation methods, electrospraying has been established as a promising and appreciable approach. Principally, it is an electrohydrodynamic processes used in the formation of nanoparticles [79]. To produce nanoparticles by electrospraying approach, high voltage electric field is applied into the polymeric solution flowing out of the nozzle in order to break it down to very fine nano-sized droplets possessing the same charge which assists their dispersion and prevents possible coagulation [80,81]. The droplet size and morphology can be tuned by altering specific variables that can be classified into the process related parameter, such as applied electric voltage, solution flow rate, distance between needle tip and collector and the type of collector. Additionally, material-related parameters are: type of polymer, its MW and concentration, as well as excipients used [82]. Chitosan nanoparticles have been recently fabricated using electrospraying technique, exhibited unique features that may have wide applications in pharmaceutical and biomedical fields (Figure 8) [83,84,85,86]. The advantages of this method can be summarised by its simple and one step technique, high MW polymers can be used, monodispersed nanoparticles can be obtained and low cost [87], however, the disadvantage is associated with the low yield product.

## 3. Insulin-Loaded Chitosan Nanoparticles

**Figure 9:**
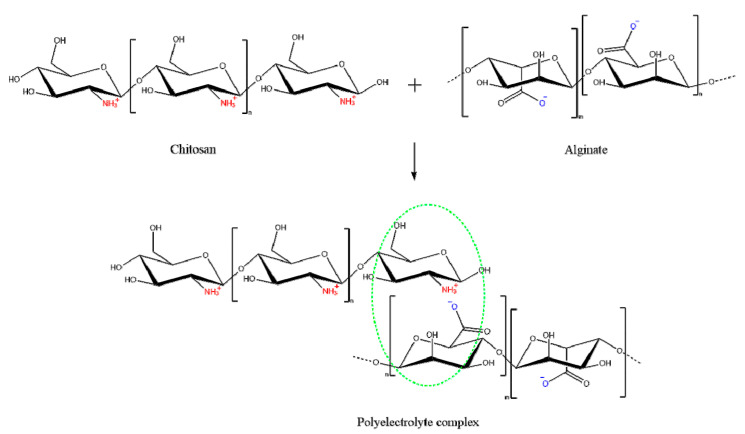
Schematic representation of the interaction between alginate as a polyanion and chitosan as polycation: mixing of the oppositely charged polyelectrolytes leads to formation of a polyelectrolyte complex caused by the electrostatic interactions between the ions.

Chitosan nanoparticles have been widely used to encapsulate insulin mainly via ionic gelation and polyelectrolyte complexation methods. This is attributed to their simplicity, and ability to produce nanoparticles under mild conditions where heat, organic solvent, or toxic cross-linking stabilizer agents are negated. Moreover, the prepared nanoparticles possess narrow size distribution [67,88]. Both ionic gelation and polyelectrolyte complexation are classified as electrostatic complexation method mainly based on the cationic nature of chitosan mediated by protonation of its amine groups in acidic medium [67]. When protonation degree of chitosan chains is adequate, it enables reactions with anionic molecules, providing excellent gel-forming properties that impart a unique opportunity for insulin entrapment [89]. The main difference between the two methods is the nature of the anionic molecules. Ionic chitosan gelation is mediated by small anionic molecules, such as phosphate, citrate, sulphate, while polyelectrolyte complexation is achieved by anionic macromolecules such as dextran sulphate, alginate, hyaluronic acid and DNA [88,90]. The latter approach is often referred to as interfacial coacervation or complex coacervation. One of the most studied polyanion with chitosan is alginate, which is a non-toxic, biocompatible and biodegradable, mucoadhesive and non-immunogenic anionic polymer (Figure 9) [91].

**Figure 10:**
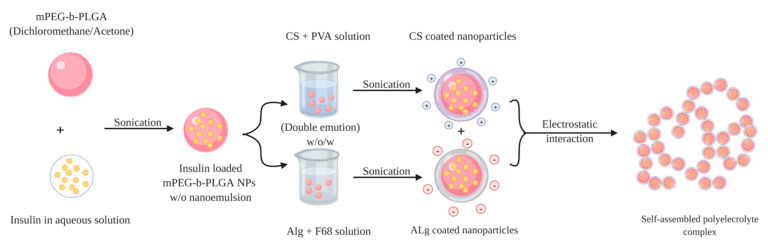
Schematic representation of the double emulsion method used to prepare insulin-loaded polyelectrolyte complexes. Positively charged chitosan nanoparticles (CSNPs) and negatively charged alginate nanoparticles (AlgNPs) are prepared, respectively, then two kinds of nanoparticles are mixed together to self-assemble forming polyelectrolyte complexes by electrostatic interaction.

Chitosan-alginate polyelectrolyte complexation can be enabled via three main approaches: ionotropic pre-gelation of alginate with calcium chloride (CaCl_2_) or any divalent ions followed by complexation, mixing diluted solutions of chitosan and alginate to acquire a plain complex coacervation, or oil-in-water (o/w) microemulsion of alginate followed by further complexation with chitosan [92]. Mukhopadhyay et al., have managed to prepare insulin-loaded core/shell chitosan-alginate nanoparticles using the first approach by admixing dropwise insulin in 0.1 M HCl and CaCl_2_ to the prepared alginate solution, forming ionic polyelectrolytes after appropriate sonication. This step was followed by chitosan polyelectrolyte complexation by adding chitosan solution with mild stirring to form core-shell nanoparticles via electrostatic interactions. The prepared nanoparticles were characterised by small particle size of 100–200 nm, high encapsulation efficiency of ~85% and pH-responsive sustained release of insulin. The oral administration of insulin-loaded nanoparticles at a dose of 100 IU/kg in the in vivo study exhibited a maximum serum insulin concentration at the 7th h of administration, advocating the nanoparticle’s ability to cross intestinal epithelium and protects insulin from enzymatic degradation in GIT. The results showed that the nanoformulation conferred significantly higher bioavailability (8.11%) than mere oral administration of insulin. Hepatotoxicity test to evaluate any possible toxicity of the nanoparticles by measuring liver-specific enzymes; alanine aminotransferase (ALAT) and aspartate aminotransferase (ASAT), has reported that neither liver was damaged nor the liver function was disrupted, demonstrating the safety of the prepared nanoparticles after oral administration [93]. Bhattacharyya et al., have applied similar preparation method as above to form core-shell nanocarrier for oral insulin delivery. In their study, instead of using mere alginate, a homogenous blend of polyurethane, a biodegradable and biocompatible synthetic polymer, with alginate (PU-Alg) was utilised to synthesise the core of the desired nanoparticles. The nanoparticles were formulated by adding a mixture solution of insulin and CaCl_2_ dropwise to PU-Alg blend solution while maintaining sonication for 15 min to allow the construction of the nanoparticle core. Then chitosan solution was added and sonicated for another 15 min to prepare PU-Alg core and chitosan shell nanoparticles. The prepared nanoparticles exhibited small particle size 90–100 nm, more than 90% encapsulation efficiency, prolonged blood glucose lowering in diabetic mice (up to 98 mg/dL for the insulin dose of 100 IU/kg at the 10th h), and relatively improved insulin bioavailability (10.36%) [94]. Polyelectrolyte complex of chitosan and alginate has been used by Chen et al., to develop a modified system as a second step after preparing insulin-loaded nanoparticles by double emulsion w/o/w solvent evaporation method. Insulin-loaded w/o/w nanoemulsion of coated chitosan and alginate were firstly prepared, then mixed together for further coacervation of polyelectrolyte complexes as an efficient oral insulin delivery vehicle (Figure 10). The encapsulation efficiency of the obtained alginate-coated and chitosan-coated nanoparticles were 81.5 ± 7.4% and 55.2 ± 7.0%, respectively and average particle size range 200–300 nm. The polyelectrolyte complex exhibited a relative bioavailability of 7.51%, non-cytotoxicity against Caco-2 cell, pH responsive propensity and controlled release profiles (sustained release at pH 6.8, while protecting the drug at pH 1.2) [95]. It could be concluded that additional polyelectrolyte complex step has brought about significant improvement on blood glucose lowering propensity by 3-folds for a duration up to 12 h compared to polyelectrolyte complex-free formulation as a result of modulating insulin release throughout the GIT.

In another study, low MW PEG (5 kDa) was conjugated with anionic polymer chondroitin sulfate and self-assembled with the cationic chitosan to render negatively charged insulin nanoparticles. In this study, Pereira De Sousa et al., aimed to prepare highly mucus-permeating nanoparticles by combining two different strategies namely, the virus-mimicking and surface PEGylation approaches. Despite of relatively large particle size (510–670 nm) obtained, the nanoparticles exhibited five-fold higher as mucus permeation enhancer as compared to non-PEGylated ones [96].

Recent study has utilised Dz13Scr, an anionic oligonucleotide with excellent biocompatibility and minimal cytotoxicity with chitosan to formulate insulin nanoparticles via complex coacervation technique. The developed nanoparticles showed acceptable size range (479 ± 24 nm), uniform polydispersity index (PDI 0.34 ± 0.06) and improved encapsulation efficiency (88.71 ± 0.3%). It was envisaged that this formulation is a potential oral insulin delivery system as it demonstrated improved stability in acidic condition mimicking those in the stomach with only 13% insulin release, while in alkaline medium, a biphasic release pattern of initial burst release (49.49%) followed by a sustained release propensity (88%) in 10 h was attainable. Moreover, the physiochemical properties of the prepared nanoparticles remained stable after being stored for two months at 4 °C as compared to newly-synthesised formulations. The developed nanoparticles achieved a balance between the mucoadhesive property caused by the cationic chitosan and the mucopenetrating capacity attributed to hydrophilic Dz13Scr presence. As a result, the encapsulated insulin was able to permeate across the GI cells (approximately 68% of encapsulated insulin translocated to the basolateral chambers within 1 h) and induces glucose consumption; as it demonstrated comparable effect in promoting the glucose uptake from 16.98% when native insulin used to 20.79% of glucose uptake in C2C12 cells by 12 h of treatment [97].

Recently, modified hybrid systems were developed to merge the conventional ionotropic gelation of chitosan with other preparation methods or strategies to obtain the most out of the prepared nanoparticles in terms of preferable characteristics, such as enhanced bioavailability of encapsulant and improved stability along GIT. Erel G et al., have designed insulin-loaded chitosan nanoparticles initially by ionic gelation between chitosan and TPP. As a novel approach, the nanoparticles were then loaded into the inner phase of prepared w/o microemulsion to grant controlled release property, enhance in vivo stability and promote drug absorption in the GIT. The effect of incorporating insulin into chitosan nanoparticle had a significant protective effect. After 8 h of administration of insulin-loaded chitosan nanoparticles embedded in microemulsion, the blood glucose level reduced by 33.6% of the initial blood glucose level, compared to only 17% reduction in the case of nanoparticles-free microemulsion [98].

Another new method was developed by He et al., also depends on the electrostatic interaction between chitosan and TPP to prepare size-controlled chitosan nanoparticles for oral insulin delivery called flash nanocomplexation (FNC). In this method, a multi-inlet vortex mixer was used to infuse aqueous solutions of chitosan, TPP and insulin to assure an efficient and rapid mixing to fabricate highly uniform insulin-loaded nanoparticles. This method enables advantage of continuous production of nanoparticles with controlled and reducible particle size (45 nm) while maintaining high encapsulation efficiency (90%), compared with the ordinary dropwise method at 92 ± 8.4 nm and 62.3 ± 4.9% for particle size and encapsulation efficiency, respectively [99].

Incorporating chitosan-insulin polyelectrolyte complex (CS-Ins-PEC) with lecithin liposomes to formulate chitosan/lithin liposomal nanovesicles was investigated by Al-Remawi et al., as a possible carrier for insulin oral delivery. Insulin was first reacted with chitosan to form Ins-CS PEC, then the PEC was added to the negatively charged liposomal dispersion developing Ins-CS PEC-associated lecithin liposomes. The optimal formulation possessed high net zeta potential around −30 mV and small particle size of 105 nm ± 17 nm when the ratios of Ins-Cs complex to lecithin was 9% (v/v). The encapsulation efficiency was slightly improved due to the presence of chitosan to interact with insulin comparable to similar chitosan-free formulations [100]. For in vivo study, blood glucose lowering effect was observed after 2 h of oral administration accompanied with a prolonged effect up to 8 h. However, the effect was modest that can be attributed to the relatively poor association efficiency [101]. Table 2 represents the most recent examples of the compositions, method of preparations and attributes of insulin loaded chitosan-based nanoparticles.

## 4. Chitosan Modification

Chitosan poor solubility in water and most organic solvents hinders its applications in various fields. One of the unique characteristics that confers chitosan a superior polymer to other polysaccharide is the ease of chemical modifications thanks to its chemical structure, especially in the C-2 position, that is prone to chemical reactions. Chemical modification is not only able to improve the physical and chemical attributes of chitosan, but also its biological characteristics. In addition, chemically modified chitosan can retain its unique properties and expand the application range of chitosan derivatives through introducing new desirable properties [103]. These derivatives have been known to have wider range of solubility compared to chitosan, can protect drugs in the acid environment, increase their release in basic medium and then their permeation (especially for hydrophilic drugs) and also can be targeted for colon-specific delivery [67]. Chitosan possesses three types of reactive functional groups, including, an amino group at the C-6 position, a primary hydroxyl group at the C-6 position and a secondary hydroxyl group at the C-3 position. The amino group at the C-6 position distinguishes chitosan from chitin with reference to its physical, chemical and biological propensities. Hydroxyl group at the C-3 position has poor rotational tendency and exhibits high steric hindrance and reduced ability to react, while modification on at the amino group at C2 or C6-hydroxyl group positions can be easily achieved [104]. The most widely employed method for chemical modulation of chitosan is N-substitution in which the amino group (–NH_2_) of chitosan poses the functional group that reacts [103].

### 4.1. Trimethyl Chitosan (TMC)

TMC is one of the most successfully developed quaternized derivative of chitosan [75]. It is advantageous in term of enhancing the paracellular transport through binding directly to negatively-charged mucus and increasing the retention time of cargo on the mucosal surface prior to opening the tight junctions of epithelial cells [105]. Many researches have synthesised TMC due to its solubility in a wide range of pH (1–9), thus regardless varying pH value along GIT, the positive charge is maintained and so the advantageous properties of the polymer [106,107].

TMC was synthesised as a nanoparticle matrix for oral insulin delivery by many experimenters to investigate its favorable characteristics compared to native chitosan. Tsai et al., have reported a potential multifunctional nanoplatform composed of TMC and fucoidan (FD), a polysaccharide that has blood glucose level regulation feature via inhibiting the activity of α-amylase and α-glucosidase enzymes. The nanoparticles have been formulated via a simple polyelectrolyte complex method between TMC and FD. The prepared nanoparticles were able to modulate the tight junction integrity and so the barrier function of the Caco-2 intestinal epithelial cell monolayer. Both TMC/FC and CS/FC enabled reduction the trans-epithelial electrical resistance (TEER) at pH 6.5. However, TMC showed its superiority at pH 6.8 and pH 7.4, as TMC/FD nanoparticles exhibited stronger effect (38.7% and 44.5% of the initial value after 120 min) than CS/FD nanoparticles on reducing the TEER. The findings indicated that by increasing the pH value, permeation enhancement ability of CS has decreased while TMC has remained unchanged. TMC/FD nanoparticles exhibited concentration-dependent α-glucosidase inhibitory activity, with an inhibition ratio of 33.2% at 2 mg/mL. In vitro release study demonstrated that TMC/FC formulation suppressed insulin release in all mediums compared to CS/FC formulation. Almost 90% of encapsulated insulin released from CS/FD at pH 6.8 vs. 35% from TMC/FD nanoparticles which may be attributed to the enhanced stability achieved by the positive charge. TMC/FD nanoparticles also demonstrated more protection of insulin against enzymatic degradation than CS/FD nanoparticles (57.8% for TMC/FD vs. 38.9% for CS/FD nanoparticles after 0.5 h of digestion). However, it appeared that TMC/FD nanoparticles are yet to improve insulin stability under enzymatic digestion for prolonged duration of 4 h (remained insulin ratio 25.6%) [105].

Another work by Omid et al., aimed to prepare an absorption-improving system of insulin-loaded nanoparticles derived from glycyl-glycine (GG) and alanyl-alanine (AA) conjugates of CS and TMC. This formulation can be absorbed by two different mechanisms: firstly, CS and its derivatives have displayed enhanced absorption from intestinal epithelium via paracellular pathways through tight junctions opening. Secondly, the conjugation of dipeptide (AA and GG) can promote the absorption as a function of proton-coupled oligopeptide transporters PepT1 and PepT2 in the cell membrane that are specific for oligopeptides with 2–4 amino acids. Thus, the formulation can induce internalisation of the conjugates through PepT1 and/or PepT2 transporters beside the tight junction opening mechanism. The optimised formulations demonstrated small particle size (157.3–197.7 nm), relatively high zeta potential (24.35–34.37 mV), and improved entrapment efficiency (70.60%–86.52%). The prepared GG- and AA-conjugated TMC nanoparticles exhibited 2.5–3.3-fold higher permeability of insulin in Caco-2 cell line compared to unmodified TMC nanoparticles. In animal model, oral administration of GG and AA conjugate nanoparticles of TMC demonstrated reasonable increase in serum insulin level (almost 50%, 45%), with relative bioavailability of 17.19% and 15.46%, respectively compared with TMC nanoparticles (14.15%) [106].

TMC, as cationic molecules, possess high affinity towards the negatively-charged mucin that forms the mucus matrix resulting in its mucoadhesive property which increases the retention time and local concentration of the nanoparticles on the mucus layer. However, entrapping within mucin matrix can decrease the direct contact with epithelial cells and increase the probability of the nanoparticle clearance together with the mucus. To circumvent this limitation, Liu et al., prepared nanoparticles of a core composed of insulin and TMC, and a dissociable “mucus-inert” hydrophilic coating of N-(2-hydroxypropyl) methacrylamide copolymer (pHPMA) derivative. Coating with HPMA copolymer on the surface of the mucoadhesive TMC nanoparticles could facilitate permeation of nanoparticles through mucus, by enabling detachment in time to expose the TMC nanoparticles, thus increasing the affinity with cell membrane and fulfilling the subsequent paracellular transport across epithelium. While oral free insulin solution failed to reduce the blood glucose level, both TMC nanoparticles and HPMA-coated TMC nanoparticles render significant hypoglycemic effect as the blood glucose level was decreased by almost 20% and 36% at 4 h, and maintained up to 10 h. HPMA-coated TMC nanoparticles at dose of 50 IU/kg, exhibited a relative bioavailability of 8.56%, which is 2.8-fold higher than that of TMC nanoparticles (3.09%) [108].

Another study by Sheng et al., also encountered similar limitation when they prepared TMC-coated polylactide-co-glycoside (PLGA) nanoparticles (TMC-PLGA NPs). The formulation was designed to overcome the multiple barriers against oral insulin absorption. Although the TMC-PLGA NPs could improve the mucus penetration of insulin in mucus-secreting HT29-MTX cells, it was noticeable that TMC-PLGA NPs moved more slowly through the GIT compared with unmodified PLGA NPs. As a consequence of slow permeability, a considerable amount of insulin might be released in the GIT and would be more susceptible to be degraded by the proteolytic enzymes [109]. Thereby, the same team suggested some modifications to the formulation to modulate the absorption of insulin released from nanoparticles in GIT via incorporating low molecular weight protamine (LMWP) as a cell penetrating peptides-linked insulin conjugates. By adopting this delivery system, the mucoadhesive nanoparticle can promote the retention on the mucus layer, while proteases degrading effect of released insulin-conjugate is avoided as a result of the short distance to the epithelia and the high permeation profile of the conjugates through epithelia. Insulin-LMWP conjugates mucoadhesive nanoparticles showed an oral insulin bioavailability of 17.98 ± 5.61%, as well as enhanced hypoglycemia effect by two-fold higher than the nanoparticles loaded-mere insulin [110].

### 4.2. Carboxymethyl Chitosan (CMCS)

Besides TMC, chitosan solubility can be improved by another common derivative via carboxymethylation, a hydrophilic modification to yield carboxymethyl chitosan (CMCS) with numerous biomedical applications. This modification enhances chitosan’s solubility in natural and basic mediums while the effect on other important characteristics is negated. Carboxymethylation reaction takes place either at C-6 hydroxyl groups or at NH_2_ moiety to produce N,O–carboxymethyl chitosan compounds that comprise primary (–NH_2_) or secondary amine group (–NH–CH_2_COOH) [111].

One of the essential applications of CMCS is a starting material to prepare nanoparticles for protein delivery such as insulin. Recently, Wang et al., have developed pH responsive nanocarrier composed of CS and CMCS for oral insulin delivery. Two insulin-loaded CMCS/CS nanogels (NGs) with similar shape, size, but opposite surface charges were prepared to investigate the effect of NGs surface charge properties on the absorption sites of small intestine. Ex vivo study exhibited unchanged adhesion and permeation propensities in the duodenum of the rat, while higher adhesion (3-fold) and permeation (1.7-fold) were observed in the jejunum for negatively charged NGs compared to the positive ones. Moreover, in vivo experiment revealed that oral administration of insulin-loaded CMCS/CS-NGs (−) enabled reduction of blood glucose level more efficiently compared to CMCS/CS-NGs (+) at 4 h (82.8 mg/dL vs. 138.6 mg/dL), and this effect was prolonged for up to 11 h [112].

### 4.3. Cyclodextrin-Grafted Chitosan

Cyclodextrins (CD) are basket-shaped molecules that possess a central hydrophobic cavity and outer hydrophilic shell. The internal hydrophobic cavity surfaces of CDs are capable to form inclusion complexes with drugs of appropriate size. Macromolecules such as insulin exemplifying too large molecules for complete inclusion, can partially be complexed by CDs via their hydrophobic side chains. Surprisingly, even if the macromolecules are partially included into the CD cavity, their physicochemical and biological properties can be significantly improved [113,114].

Chitosan and CD were integrated together to prepare carboxymethyl-β-CD-grafted CS nanoparticles (CMCD-g-CS nanoparticles) via ionic gelation method [113,115]. Song et al., have synthesised CMCD-g-CS by EDC-mediated esterification reaction, then TPP solution was gradually added into the insulin/CMCDg-CS solution for 1 h under continuous stirring. The prepared nanoparticles exhibited favourable encapsulation efficiency (57.0 ± 1.38%). The in vitro release experiment demonstrated that CMCD-g-CS nanoparticles could efficiently protect encapsulated insulin in acidic medium (only 35.4% insulin released in SGF) that may account for the free insulin located at the nanoparticle surfaces. A greater extent and higher insulin released was attainable in SIF with a cumulative amount higher than 80% over 1.5 h dissolution, achieving bioavailability of 14.54%. Insulin-CMCD-g-CS nanoparticles were advocated to be highly biocompatible with minimised cellular toxicity effects, where more than 80% cell viability has reported after incubating in nanoparticles solution at a concentration range of 20–120 µg/mL [115].

### 4.4. Vitamin B12-Conjugated Chitosan (VitB12-Chi)

Layer-by-layer (LBL) approach that comprises polyelectrolytes of opposite charges and placed successively onto core surface, have exhibited significant influence on physiochemical and therapeutics attributes of the prepared nanosystem. This method was used to fabricate insulin nanoparticles for oral delivery under mild conditions to avoid protein decomposition by organic solvents or high temperature. Nevertheless, LBL method is a lengthy process and involves repeated adsorption and centrifugation steps that may hinder effective encapsulation. Implementation of vitamin B12 as a transport system has exhibited great interest as a potential approach to promote insulin absorption in GIT and improves its bioavailability [37]. Su et al., have fabricated insulin nanoparticles via LBL technique and conjugated vitamin B12 with chitosan and alginate to produce a nanocomplex (VitB12-Chi-CP nanoparticles). This nanocomplex has formed a complex with intrinsic factor (IF) that subsequently incurred receptor-mediated endocytosis through intestinal epithelia. VitB12-Chi-CP complex has enhanced the water solubility at higher pH values compared to vitamin B12 free nanoparticles. It was also found that 14% of insulin has been transported through Caco-2 cells monolayer upon incubation with nanocomplex for 6 h that could be attributed to endocytosis uptake of vitamin B12 and paracellular passage. Moreover, vitB12-Chi-CP nanoparticles also possessed low zeta potential that gave rise to thinner coating formation and thus reduced particle size. In addition, prolonged blood glucose lowering profile was attainable for up to 12 h, despite the delayed onset of action by 1–2 h [116]. Table 3 illustrates the recent examples of nanoparticles-based modified chitosan matrices, their compositions and characterizations.

## 5. Chitosan and Chitosan Derivatives as Coating Material for Insulin Nanoparticles

In the field of drug delivery by means of nanotechnology, chitosan has not only been widely used as a particle-forming polymer, but also as a surface coating. Chitosan as a coating material can be used with different types of nanoparticles such as, polymeric nanoparticles, lipid nanoparticle and metal-based nanoparticles. Many advantages can be obtained through nanoparticle’s surface modulating by means of coating with chitosan, including, drug release and stability control, bioavailability improvement of drug loaded, mucoadhesive enhancement and tissue penetration property [118].

Chitosan and chitosan derivatives have been used in several studies as a coating material to protect insulin-loaded nanoparticles, as it is believed to have a vital role in protecting insulin from premature release in the gastric medium and increase the residence time of nanoparticles at the intestinal mucosa upon reaching the small intestine [119]. Chitosan coated nanoparticles can be prepared by either addition of chitosan solution into previously prepared nanoparticle formulations, an easy and frequently described way to coat nanoparticles, or by adding chitosan solution during the nanoparticle formation process.

Lopes et al., prepared insulin-loaded alginate/dextran sulfate nanoparticles coated dually by chitosan and albumin, in order to enhance the permeability through the intestinal epithelial layer. The dual coated nanoparticles have resulted in a higher permeability of insulin across Caco-2/HT29-MTX/Raji B cell monolayer compared to coat-free ones. Drug dissolution results exhibited reduced insulin release (30%) in the gastric medium followed by sustained release in the intestinal medium [120]. The second approach is by adding chitosan or chitosan derivative solution during the formation process usually produces so-called core-shell nanoparticles.

As for lipid nanocarriers, chitosan is considered one of the best choices among various polysaccharides as coating materials, because of its positive charge and high reaction tendency with negatively charged liposomal surfaces, thus ensuring firm coating. Chitosan-coated liposomal surface could enhance the stability and bioavailability of liposomes [121]. Shalaby and El-Refaie have prepared chitosan-coated insulin-loaded cationic liposomes. The chitosan coated liposomes exhibited nano-sized at 439.0 ± 12.3 nm and zeta potential at +60.5 ± 1.9 mV. Chitosan-coated insulin-loaded cationic liposomes improved insulin loading efficiency (87.5 ± 0.6%), with prolonged pharmacological effect as the ex vivo intestinal mucoadhesion showed increased tissue residence of chitosan-coated compared to uncoated liposomes. Premature insulin released in simulated gastric fluid was minimised (18.9 ± 0.35%), while high amount (73.33 ± 0.68%) was released in simulated intestinal fluid over 48 h dissolution [122].

In another study, Moghassemi et al., prepared insulin-loaded niosomes by the reversed-phase evaporation method followed by TCM coating via incubating the suspension in a polymer. The investigation of the ability of TMC-coated niosomes in facilitating the permeability of insulin through Caco-2 cell monolayer, showed that TMC-coated niosomes enabled opening the cells tight junctions of the intestinal membrane model and facilitated the enteral absorption of insulin by 4-folds as compared to insulin alone [123]. Table 4 illustrates the recent examples of chitosan and chitosan-derivatives as coating materials.

## 6. Conclusions

This review demonstrated that extensive research activities have been devoted on the application of insulin nanoparticles-based chitosan/chitosan derivatives. Successful loading of insulin and the ability of modulating the size, surface charge, and insulin release confers chitosan nanoparticles a promising tool for oral insulin delivery. Verities of method preparations could impart a desired attribute to the prepared nanoparticles. Compared to sole oral administration of insulin, chitosan nanocarriers have significantly improved the bioavailability and blood glucose lowering propensities. This is mainly attributed to the mucoadhesion characteristic of chitosan to prolong the residence time of the nanoparticle near to the absorption sites in the intestinal region and facilitate insulin transport either transcellularly or intracellularly to blood capillaries, heading to the liver via the portal vein. Chitosan derivatives are introduced to improve chitosan solubility and widen its application as a drug delivery system, while maintaining its useful properties. Also, modifying chitosan could have potential applications to control cargo release throughout GIT and surface charge of the prepared nanoparticles to further protect and promote insulin absorption, and hence the bioavailability. In addition, chitosan/chitosan derivatives can be applied as a coat for nanoformulation as an additional use to meet a specific desired goal.

## Figures and Tables

**Table 1:** Effects of different barriers in gastrointestinal tract (GIT) against insulin oral delivery.

Barriers against Oral Insulin Administration				
Physical Barriers			Chemical Barriers	Enzymatic Barriers
**Mucus layer**	**Epithelial layer** **(Trans-cellular transportation)**	**Tight junctions** **(Para-cellular transportation)**	Stomach: highly acidic (pH 1–3.7) ↓ Denaturation and degradation of insulin. Intestine: neutral and slightly alkaline (pH 6–8).	Insulin breakdowns by the protease’s enzymes found in the GIT [24]. Stomach: pepsin. Intestine: mainly trypsin, chymotrypsin
Viscous, hydrophilic, negatively charged layer ↓ Permitting only hydrophilic net-neutral molecules to pass ↓	Highly limited to lipophilic drugs with molecular weight less than 700 Da as the membrane is mainly consisting of phospholipid bilayers [30].	Regulate the transportation of molecules in between the epithelial cells.		
Hydrophobic drugs and proteins are unable to cross, while cationic compounds exhibit low diffusion rate than neutral ones [31,35].	Insulin is hydrophilic protein with high molecular weight 5800 Da.	Selectively permeable to small hydrophilic molecules [30,34,36].	This variation in pH values may cause pH-induced oxidation and deamination of the protein [37,38].	cytosolic and membrane-bound enzymes in the microvilli of intestinal enterocytes [39,40].

**Table 2:** Examples of chitosan-based nanoparticles-loaded insulin.

**Nanocarrier**	**Preparation Method**	**Particle Size (nm)**	**Zeta Potential (mV)**	**Entrapment Efficiency (%)**	**In Vitro Insulin Release**	**Dose (IU/kg)**	**In Vivo Observation**	**Reference**
Chitosan (CS) MW (25–65 kDa), 83–86% Deacetylation Degree(DD) + Alginate (ALG) MW (1.03 × 105 g/mol)	Polyelectrolyte complexation	216	+3.89	78.3	A burst release with max. of 26.7% of insulin release was found in pH 1.2, followed by a sustained and prolonged insulin release (79–84%) through 24 h.	Oral: 50–100 SC: 5	Insulin-loaded CS/ALG NPs (50 and 100 IU/kg) showed reduction in the blood glucose level to 143 and 104 mg/dL, respectively, with sustained effect up to 9 h.	[93]
Medium MW, 75%, 85% deacetylated Chitosan + TPP ratio 6:1	Ionic gelation method	Nanoparticle 356.5 ± 43.4 (Microemultion) 99.1 ± 28.7	Nanoparticle 46.5 (Microemultion) 13.1	-	At pH 2.5 after 2 h, insulin release from microemulsion was 48.1%. At pH 6.8 after 2 h, the release was 51.2% and after 3 h it was 66.1%.	Oral: 50 SC: 1	Plasma glucose level reduced to 68.7% after 3 h and it maintained at 66.4% of the initial blood glucose level after 8 h.	[98]
Chitosan 25 kDa, + Chondroitin sulphate (ChS) 20–30 KDa + Polyethylene glycol 5000 Da (PEG)	Ionic gelation	510–670	−1 to −5	2.18 ± 0.70	In simulated intestinal fluid (SIF) buffer, insulin release profile showed a gradual release of the protein reaching 65% in 4 h, followed by a plateau.	-	-	[96]
90 KDa MW, 85% deacetylated chitosan + TPP	Flash nanocomplexation using multi-inlet vortex mixer	46.2 ± 2.7	9.4 ± 1.2	91.0 ± 1.7	The amount of released insulin at pH 2.5 was about 16%, while negligible amount at pH 6.6, and a sustained release of insulin within a few hours at pH 7.4	Oral: 60 or 120 SC: 10	Gradual but distinct reduction of blood glucose levels by 51% (60 IU/kg) and 59% (120 IU/kg) within 8 h.	[99]
Chitosan (28 kDa) + Lecithin liposomes + L-Arginine	CS-insulin dispersion (polyelectrolyte complexation) added to lecithin liposomal dispersion	105 ± 17	−30	20	Insulin was rapidly released in both 0.1 M HCl and phosphate buffer pH 6.8 media and complete release was achieved almost after 30 min.	Oral: 50 SC: 1	A significant effect was observed at 2 h after oral administration as the blood glucose level was reduced by almost 17% of the initial level and the effect was prolonged for up to 8 h.	[101]
Low MW 50–190 kDa, ≥75.0% deacetylated chitosan + Iota-carrageenan (CMCi)	Polyelectrolyte complexation method	613 ± 41	52.5 ± 0.5	86.9 ± 2.6	After 2 h in simulated gastric fluid (SGF), the release of insulin from the nanoparticles was only 4.91 ± 0.24%, while in SIF, the release of insulin was 86.64 ± 2.20%.	-	-	[102]
Chitosan, alloxan monohydrate + Alginate + Polyurethane (PU-ALG/CS NPs)	Polyelectrolyte complexation method	90–110	38.5	90	There was a slight insulin release (13.7%) at pH 1.2 up to 1 h, while moderately release (up to 50%) till 10th h in pH 6.8 buffer solution, whereas sustained release of insulin was noticed at pH 7.4 from 11th h, and reached the maximum insulin release after 20th h (98.32%).	Oral: 50 and 100 SC: 5	Blood glucose level was reduced up to 98 mg/dL for the insulin doses of 100 IU/kg, and 131 mg/dL for the 50 IU/kg dose at the 10th h.	[94]
Chitosan 95% DD + Alginate + Methoxypolyethylene glycol (mPEG, MW 5.0 kDa) + D, L-Lactide (LA) + Glycolide (GA) + Poly (vinyl alcohol)1788 low-viscosity (PVA) + poly (ethylene glycol)-block-poly (propylene glycol)-block-poly (ethylene glycol) (F68, Mw 8.4 kDa)	Double-emulsion (w/o/w) solvent evaporation method + Polyelectrolyte complexation	CS NP 224.4 ± 13.8 Alg NP 260.1 ± 17.1	CS NP +13.7 ± 1.6 Alg NP −55.7 ± 6.6	CS NP 55.2 ± 7.0 Alg NP 81.5 ± 7.4	The insulin loaded PEC enabled a slight insulin release (only 13.91%) in SGF (pH 1.2) within the first 4 h. In contrast, rapid rising rate in the first 4 h (38.03%) at the pH 6.8 took place, and the cumulative drug release increased to 51.57% within 10 h, and reached 80.54% after 60 h.	Oral: 60 SC: 5	The blood glucose level decreased after the oral administration of insulin-loaded PEC with the maximal blood glucose reduction of 30% at 8 h, and 20% after 12 h. Insulin concentration in plasma was increased gradually and resulted in a maximum plasma concentration (41.5 ± 4.4 μIU mL^−1^) at 10 h.	[95]
Chitosan (95% deacetylated; MW 150 kDa) + Dz13Scr	Complex coacervation	534 ± 24	14.57 ± 1.1	79.96 ± 3.96	Only 14.03% of cumulative insulin released at pH 2, while approximately 85% of insulin was released after 10 h at pH 6.8 phosphate buffer solution.	-	-	[97]

**Table 3:** Insulin-loaded chitosan derivatives-based nanoparticles.

Polymer	Nanocarrier Components	Method of Preparation	Particle Size, Zeta Potential	Encapsulation Efficiency, Drug Loading	In Vitro Insulin Release	Dose	In Vivo Observation	Reference
Trimethyl chitosan	400 KDa MW, >90% deacetylated TMC+TPP + Poly N-(2-hydroxypropyl) methacrylamide (HPMA) (pHPMA)	Mild electrostatic self-assembly process	163.1 nm −3.35 mV	54.1 ± 1.9% 26.5 ± 0.7%	Rapid insulin release at pH 2, while within 8 h, sustained release was observed at both pHs 6 and 7.4. 70% of insulin released in presence of trypsin within 4 h.	Oral: 50 IU/kg SC: 5 IU/kg	36% decreasing of blood glucose level (BDL) at 4 h, and the effect lasted for 10 h.	[108]
Trimethyl chitosan	275 KDa MW, 95% deacetylated chitosan (CS)/(TMC) + Alanyl alanine (AA) + Glycyl-glycine (GG)	Polyelectrolyte complexation	CS-GG 167.8 ± 46.1 nm 25.40 ± 4.2 mV CS-AA 185.3 ± 27.6 nm 24.62 ± 3.6 mV TMC-GG 157.3 ± 38.5 nm 34.37 ± 5.1 mV TMC-AA 197.7 ± 31.7 nm 24.35 ± 1.9 mV	CS-GG 86.52 ± 4.7% 56.81 ± 6.7% CS-AA 77.20 ± 5.9% 30.92 ± 4.6% TMC-GG 70.60 ± 7.2% 39.07 ± 2.6% TMC-AA 83.08 ± 6.2% 37.24 ± 1.6%	Burst release of insulin within the first 30 min, after that insulin has been released in a controlled manner and reached a maximum of 83.4% in CS-GG, 78.3% in CS-AA, 75.9% in TMC-GG, 73.9% in TMC-AA.	Oral: 20 IU/kg SC: 3 IU/kg	Both TMC-GG and TMC-AA nanoparticles reduced the BGL considerably compared to oral insulin. While TMC-nanoparticles decreased the BGL to only 61.3% of the initial, TMC-GG showed maximum reduction to 46.8%, followed by TMC-AA to 54% after 8 h.	[106]
Trimethyl chitosan	33 KDa MW, 85% deacetylated TMC + Fucoidan (FD) MW (31.7 kDa)	Simple polyelectrolyte complex	256.7 ± 4.9 nm 26.5 ± 1.1 mV	56.4 ± 4.3% 8.6 ± 2.2%	At pH 2 slow insulin release at 38.3 ± 2.1% and 45.2 ± 2.7% of TMC/FD and CS/FD, respectively, while at pH 7.4 the release of insulin was faster, and more rapid 75.4 ± 2.2% and 93.4 ± 1.6%, respectively.	-	-	[105]
Carboxymethyl chitosan (CMCS)	400 KDa MW, 95% deacetylated chitosan	Simple ionic gelation	CMCS/CS-NGs (−) 243 ± 3.85 nm −15.9 ± 0.45 (+) 260 ± 4.47 nm +17.2 ± 0.49 nm	CMCS/CS-NGs (−) 73 ± 6.36% 29 ± 3.61% (+) 74 ± 8.36% 27 ± 4.04%	Insulin released was 28% in SGF and approximately 87% in SIF.	Oral: 50 IU/kg SC: 5 IU/kg	At 4 h, the nanoparticles with negative charge have made BGL dropped to 82.8 mg/dL while positive ones to 138.6 mg/dL, and this effect prolonged for 11 h	[112]
Carboxymethyl chitosan (CMCS)	400 KDa MW, 95% deacetylated chitosan	Simple ionic gelation	-	-	CMCSNP (−): pH 1.2 (20.7% at 2 h), pH 7 (83.4% at 2 h) CMCSNP (+): pH 1.2 (33.6% at 2 h), pH 7 (71.6% at 2 h)	Oral: 50 IU/kg SC: 5 IU/kg		[117]
Carboxymethyl-β-cyclodextringrafted chitosan (CMCD-g-CS)	MW 46K with 90–95% deacetylation + carboxymethyl-β-cyclodextrin MW 1591 + TPP	Ionic gelation	218 nm	EE 57.0 ± 1.38%	About 35.4 ± 0.025% of insulin was rapidly released in SGF (pH 1.2) after 15 min, while in SIF (pH 7.4), after 120 min the cumulative amount of insulin released increased to 82.9 ± 0.04%.	Oral: 50 IU/kg SC: 5 IU/kg	Insulin/CMCD-g-CS nanoparticles administration showed gradually enhanced hypoglycemic effect. After 12 h, the BGL was reduced to 51.22% of the initial level. The nanoparticles exhibited a relative bioavailability of 14.54%.	[115]
Vitamin B12-grafted chitosan	Vitamin B12-grafted chitosan (75–85% Deacetyld, 65–95 kDa) + alginate + calcium phosphate	Micro-emulsion method	234.83 nm 32.56 mV	75.16% 7.83%	At pH 1.2 only 9.9% insulin was released at 2 h.	Oral: 50 IU/kg SC: 5 IU/kg	BGL reduced to 197 mg/dL and maintained up to 12 h.	[116]

**Table 4:** Examples of chitosan and chitosan-derivatives as coating materials.

Type of Chitosan	Type of Nanoparticles	Preparation Method	Observation	Reference
Chitosan (30 KDa low MW with 85% DD)	Liposomes	Simple thin-film hydration technique	In vitro: at pH 1.2, lower percentage of insulin released from CS-coated liposomes (18.9 ± 0.35%) compared to (35.9 ± 0.75%) uncoated ones after 48 h. At higher pH (7.4), CS-coated liposomes gradually released almost 74% of insulin over a prolonged time of 48 h. In vivo: Blood glucose level remarkably decreased after 1 h of CS-coated liposomes administration. BGL continued lowering until reached its normal level and maintained it for 4 h (8 h from administration).	[122]
Trimethyl chitosan (TMC) (low MW)	Niosomes	Reversed-phase evaporation method	In vitro: the insulin release rate was significantly slower than insulin alone as after 5 h, insulin reached its maximum level (12% from TMC-coated niosomes while 63.42% from free solution). TMC-coated niosomes continued to improve insulin transport until 120 min through Caco-2 cells. Insulin permeation coefficient increased by 4 folds from coated niosomal nanoparticles more than insulin alone.	[123]
Chitosan (50 kDa, 85% DD)	Solid lipid nanoparticles	w/o/w emulsion method	In vitro: CS-coated solid lipid nanoparticles demonstrated better permeation-enhancing properties as compared to the uncoated ones through Caco-2 cell monolayer. In vivo: CS-coated solid lipid nanoparticles increased the hypoglycemic effect and enhanced the pharmacological availability to 17.7% compared to 5.1–8.3% of uncoated solid lipid nanoparticles.	[124]
TMC (85% DD, degree of trimethyl substitution 50%)	Polymeric nanoparticles (PLGA-NP)	Double-emulsion solvent evaporative method	In vitro: compared with PLGA nanoparticles, TMC-PLGA nanoparticles relatively protected insulin from enzymatic degradation in the GIT. TMC-coated nanoparticles showed stronger mucoadhesive and mucus-penetrating capacity through HT29-MTX cells. The cellular uptake of insulin of TMC-PLGA nanoparticles was dramatically higher than uncoated PLGA nanoparticles through HT29-MTX cells without a mucus layer, while the amount of insulin penetrated the mucus layer was 2 folds greater for the coated TMC-PLGA nanoparticles. In vivo: 40% of TMC-PLGA nanoparticles could attach to the lower part of the small intestine prolonging the retention time at the absorption site while most of the PLGA nanoparticles moved to the colon within 3 h. TMC-PLGA nanoparticles decreased the BGL rapidly to 70% of the initial level after 7 h, and continued to decrease over 12 h. TMC coated nanoparticles also had higher pharmacological availability of 11.82% compared to 5.93% of uncoated ones.	[109]
TMC (85% DD, degree of trimethyl substitution 50%)	Polymeric nanoparticles (PLGA-NP) + (LMW protamine conjugated insulin)	Ultrasound sonication, double emulsion	In vitro: the mucoadhesive TMC-coated PLGA nanoparticles gave effective protection to encapsulated insulin or insulin-LMW protamine as only 5% of insulin released after 1 h at SGF of 1.2 pH, while 40% at SGF with pepsin compared to 90% digestion of insulin or insulin-LMW protamine in enzyme-containing SGF within 5 min. Coated nanoparticles significantly improved insulin permeability through Caco-2 cells. Insulin permeation coefficient of insulin-LMW protamine coated nanoparticles was 10-fold higher than that of insulin solution. In vivo: pharmacological availability has been remarkably enhanced (17.98%) from insulin-LMW protamine coated nanoparticles, compared to 0.91 of the free insulin-LMW conjugates.	[110]
